# Myo-inositol supplementation in gestational diabetes mellitus: is there any interference with diet?

**DOI:** 10.3389/fnut.2025.1623699

**Published:** 2025-09-24

**Authors:** Salma H. Ahmed, Ibrahim Ibrahim, Hala Abdullahi, Wided Khamlaoui, Chinnu George Samuel, Fariada Badri, Fatima Ahmad, Gbemisola Okunoye, Annalisa Terranegra

**Affiliations:** ^1^Precision Nutrition Unit, Translational Medicine Department, Sidra Medicine, Doha, Qatar; ^2^Department of Endocrinology, Sidra Medicine, Doha, Qatar; ^3^Department of Obstetrics, Sidra Medicine, Doha, Qatar; ^4^Clinical Trial Office, Sidra Medicine, Doha, Qatar; ^5^College of Health and Life Sciences, Hamad Bin Khalifa University, Doha, Qatar

**Keywords:** gestational diabetes mellitus, pregnancy, Myo-inositol, maternal diet, lifestyle

## Abstract

**Background/objectives:**

Gestational Diabetes Mellitus (GDM) affects 31.6% of pregnant women in Qatar. Myo-inositol (MI) supplementation has been proposed to reduce GDM risk, but its interaction with diet and lifestyle remains unclear. This study assessed the effects of physical activity and diet on MI supplementation and GDM onset throughout pregnancy.

**Subjects/methods:**

A randomized double-blind clinical trial was conducted at Sidra Medicine hospital, involving pregnant women assigned to either the MI (*n* = 31) or placebo (PLA, *n* = 33) arm. The Oral Glucose Tolerance Test (OGTT) was conducted between 24 and 28 weeks of gestation. Dietary intake was assessed using 24-h dietary recall and Food Frequency Questionnaires (FFQ), and physical activity and lifestyle data were collected using questionnaires at each trimester. Nutrient analysis was performed using Nutritionist Pro, and dietary patterns were evaluated using the Healthy Eating Index (HEI) and Dietary Inflammatory Index (DII). Statistical analysis was conducted using Prism GraphPad (*p* ≤ 0.05). ISRCTN Registration number ISRCTN16448440.

**Results:**

Dietary adjustments during pregnancy included increased protein and fiber intake, reduced salt, and higher consumption of simple sugars. The MI group showed higher physical activity (walking time/week), lower weight gain, and increased fiber intake (beans and tubers) compared to PLA, which consumed more high-fat, high-sugar foods. Despite these differences, no major differences in GDM incidence were observed between groups.

**Conclusion:**

MI supplementation was associated with a healthier diet and higher physical activity. Findings suggest that an active lifestyle and balanced diet may enhance MI’s efficacy in lowering GDM risk. Further research is needed to clarify this relationship.

**Clinical trial registration:**

The study Clinical trial registration ID is ISRCTN Registration number ISRCTN16448440.

## 1 Introduction

Gestational diabetes mellitus (GDM) is a prevalent metabolic condition that occurs during pregnancy. It is characterized by carbohydrate intolerance detected during pregnancy (1), associated with one or more of the following risk factors: maternal age, maternal body mass index (BMI), ethnic background, family history, previous history of GDM, and previous/current adverse pregnancy outcome (2). Diabetes is considered a public health concern in Qatar, with a rising prevalence, including GDM, which reached an incidence rate of 31.6% (3). During pregnancy, GDM is linked to a higher likelihood of experiencing pre-eclampsia, pre-term labor, Cesarean-section delivery, macrosomia, and shoulder dystocia (4). Long-term effects are also common for GDM patients. A Danish study revealed that 21% of individuals who were exposed to GDM during pregnancy developed type 2 diabetes mellitus (T2DM) by the age of 18–27 years (5). While another American study done on the Latino population in the USA, showed approximately 60% of women with a medical history of GDM develop T2DM later in life (6). In the past decade, evidence-based lifestyle interventions have been implemented to improve pregnancy outcomes in GDM patients, including personalized nutrition plans, medications and targeted physical activity programs (7, 8), all aimed at better controlling blood glycemia and improving the overall health of pregnant women. Research has demonstrated that both medications and lifestyle interventions are successful in delaying or preventing the occurrence of diabetes in women with a history of GDM (9). Although there has been notable advancement, certain obstacles persist with current interventions, such as non-adherence to dietary recommendations and mothers’ hesitance to take metformin tablets or use insulin injections. These challenges emphasize the significance of implementing new evidence-based preventative interventions. Inositol has been suggested as a dietary supplement that could potentially decrease the occurrence of GDM in pregnant women who are at a higher risk. Myo-inositol (MI), which is an isomer of inositol, is a naturally occurring monosaccharide frequently present in meat, corn, cereals, and legumes, and is categorized as a nutritional supplement by the US Food and Drug Administration. It serves as an intracellular mediator in the insulin signaling pathway and improves the body’s response to insulin. MI was associated with insulin sensitivity improvement and a lower blood sugar level in metabolic conditions, including T2DM in polycystic ovarian syndrome (10, 11). MI is a novel and safe supplement that can effectively prevent GDM by regulating maternal blood glucose levels without causing harm to the mother or fetus (12, 13). Using MI as a dietary supplement appears promising to prevent GDM and its associated complications. Multiple clinical trials have explored the effects of MI supplementation in preventing GDM. Based on the systematic review of four studies, the possible positive impact on enhancing insulin sensitivity indicates that it could be valuable in preventing GDM (14). While MI exhibits the potential to prevent GDM, there is still insufficient evidence to justify its regular utilization. Larger multicenter studies are required to evaluate the routine use of MI despite positive results mainly from smaller trials. The effect of external factors, such as individual dietary habits and lifestyle, should be taken into account while prescribing MI. To date, there’s no clear understanding of the role of diet and lifestyle in masking or boosting the effect of inositol. Comparing MI with a placebo in double-blind, randomized controlled trials, taking into account the effect of diet and exercise, will provide clearer insights into its effectiveness. Therefore, this study aims to investigate the impact of physical activity and dietary intake on MI supplementation versus placebo in pregnant women at risk of GDM.

## 2 Methods

### 2.1 Study population

Pregnant women recruited from Sidra Medicine (ISRCTN Registration number ISRCTN16448440) were recruited to conduct a randomized double-blinded clinical trial. The MIGDM study design and population characteristics were published previously (15). Briefly, pregnant women attending the antenatal care clinic at Sidra Medicine before 16 weeks of gestation and older than 18 years were considered for participation in the study after receiving comprehensive written and oral information in both English and Arabic.

### 2.2 Intervention

Randomization was done via computer-generated numbers to assign participants to MI or Placebo (PLA) arm, the details of which were previously published (15). Briefly, MI and PLA were arranged at the source with identical packaging and provided through the hospital pharmacy. The MI pack contained 2 g of Myo-inositol, whereas the placebo option contained a pharmacologically passive substrate. Participants in both arms received twice-daily dosing and completed at least 12 weeks of intervention prior to undergoing the Oral Glucose Tolerance Test (OGTT). The OGTT was performed at 24–28 weeks’ gestation for routine screening for GDM. Women were instructed to continue consuming the trial packs and OGTT results should not have an impact on the intake. Patient demographics and clinical parameters were collected, including age at the recruitment, pre-pregnancy weight and BMI categories (underweight <18.5 kg/m^2^, normal weight 18.5–24.9 kg/m^2^, overweight 25–29.9 kg/m^2^, obese ≥30 kg/m^2^), overall weight gain, fasting glucose, fasting insulin, 1-HR and 2-HR glucose post-OGTT, Homeostasis model assessment of beta cell function (HOMA-B), and Homeostasis model assessment of insulin resistance (HOMA-IR). Pregnancy and delivery outcomes were collected by the research coordinator.

### 2.3 Diet assessment

The dietary consumption was assessed through the 24-h dietary (24HR) recalls and using Food Frequency Questionnaires (FFQ) during recruitment (baseline) and the treatment at all trimesters of pregnancy. Nutrient intake was calculated using dedicated software (Nutritionist Pro, Axxya). Physical activity and Lifestyle questionnaires were administered at the same time points by a trained dietitian, including questions on walking times/week and vigorous and moderate exercise times/week, screen time/week, and sleeping hours/day.

Dietary pattern analysis was done using two scores. The first one is the healthy eating index (HEI), which reflects the diet’s alignment with key dietary recommendations. HEI utilizes 13 Food components that are weighted at 5 or 10 points according to the food type. A higher total HEI score indicates a diet that aligns better with dietary recommendations (16). The second dietary score is the dietary inflammatory index (DII), which categorizes diets based on their inflammatory effect. It is calculated by using 28 food parameters that are weighted based on the world average and standard deviation. The higher total DII score indicates that the participant is following a pro-inflammatory dietary intake and vice versa (17).

### 2.4 Statistics

Proportions and frequencies were utilized to report qualitative results, while means, median, standard deviation and interquartile range (IQR) were used to report quantitative variables. Normality tests have been performed on continuous variables; nonparametric tests have been used with not-normally distributed data, while *t*-test and one-way ANOVA have been applied for normally distributed variables. Chi-squared analysis was used to assess the differences between qualitative variables. Also, analyzing the data using logistic regression analysis to assess odds ratios (ORs) and to study the predictive effect of all risk factors. 95% confidence intervals of ORs for related factors were estimated. All statistical assessments were performed on SPSS package version 29 (IBM) and Prism Graph Pad 10, using two-sided measurements and considered significant at a *p*-value < 0.05.

## 3 Results

### 3.1 Study population and clinical data

The patients who completed the study procedures (*n* = 72) were included in the analysis for both MI (*n* = 37) and Placebo (*n* = 35) groups. Clinical parameters recorded from patients include age, pre-pregnancy BMI, overall weight gain, fasting glucose, 1-h and 2-h post-OGTT glycemia, fasting insulin, HOMA-IR, and HOMA-B measured at 24–28 weeks of gestation. The values for each arm of the intervention are summarized in Table 1. The comparison analysis revealed only a minor difference in fasting glucose in OGTT between the two groups.

**Table 1:** Clinical characteristics of study participants.

Characteristics	MI arm (Mean ± SD)	PLA arm (Mean ± SD)	*p*-value
Age at enrollment (years)	34.89 ± 1.1	34.58 ± 1.03	0.316
Pre-pregnancy BMI (kg/m^2^)	28.1 ± 1.1	26.1 ± 0.9	0.340
Overall weight gain (kg) according to pre-pregnancy BMI			
Underweight	NA	NA	NA
Normal weight	10.2 ± 2.3	11.9 ± 3.2	0.168
Overweight	5.5 ± 5.6	3.5 ± 7.1	0.482
Obese	2.8 ± 19.4	16.7 ± 2.3	0.286
Fasting glucose in OGTT (mmol/L)	5.03 ± 0.1	4.9 ± 0.1	0.032
Glycemia 1 h post OGTT (mmol/L)	7.4 ± 0.6	8.2 ± 0.5	0.464
Glycemia 2 h post OGTT (mmol/L)	6.8 ± 0.6	7.03 ± 0.5	0.791
Fasting insulin (mU/L)	7.9 ± 1.1	7.7 ± 0.9	0.150
HOMA-B	100.5 ± 12.6	110.2 ± 16.4	0.772
HOMA-IR	1.8 ± 0.3	1.7 ± 0.2	0.086

BMI, Body mass index; NA, Not applicable; OGTT, Oral glucose tolerance test; HOMA-B, Homeostasis model assessment of beta cell function; HOMA-IR, Homeostasis model assessment of insulin resistance.

**Figure 1:**
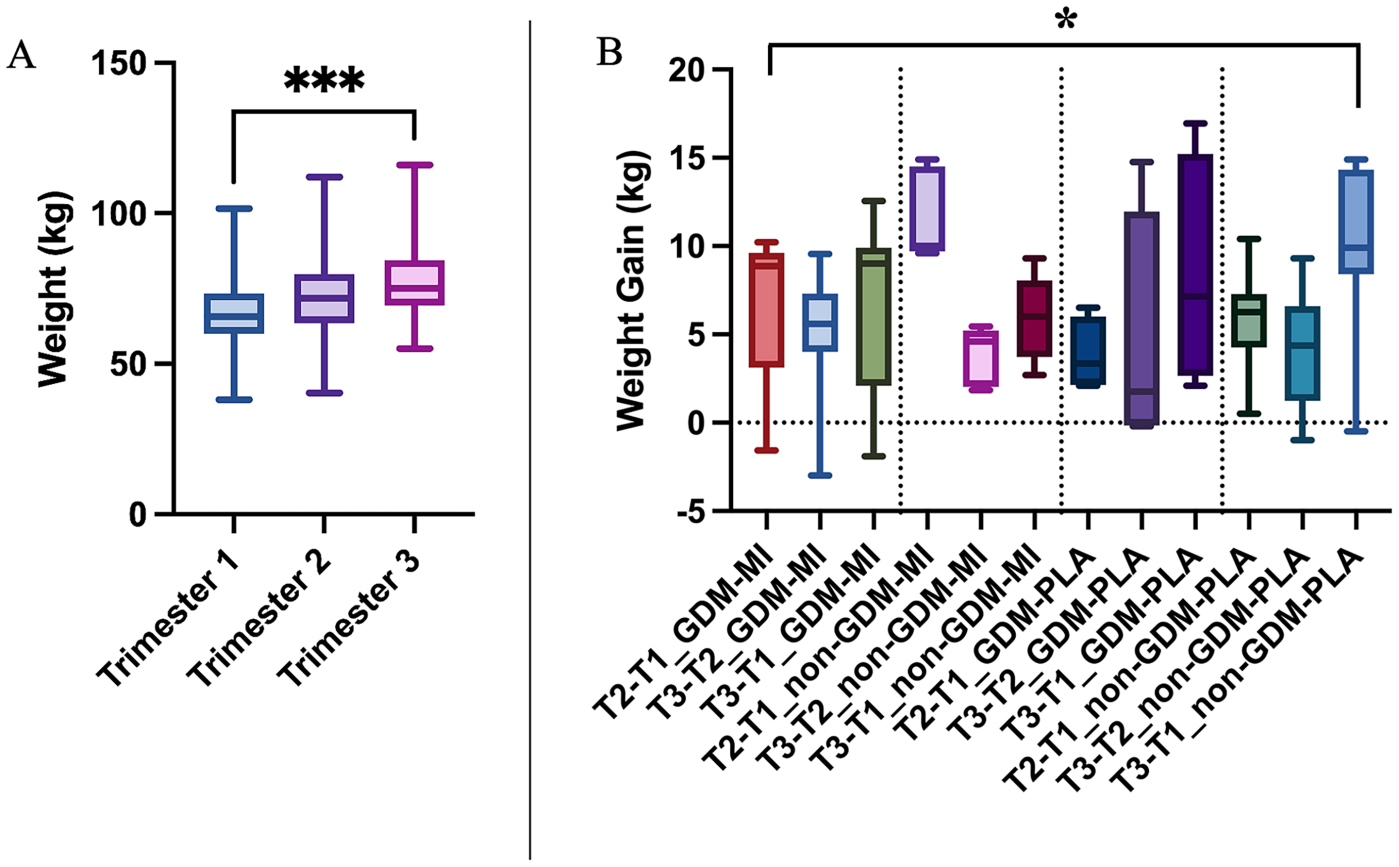
**(A)** Weight gain comparison across trimesters of pregnancy by using the Kruskal–Wallis test; and **(B)** Weight gain across treatment and GDM groups by using the One-way ANOVA test; *p < 0.05; ***p < 0.001.

Gestational weight increased steadily across trimesters (Figure 1A), peaking in the third trimester as expected, but showing a significant difference between MI and PLA groups and a notable weight increase in both GDM-PLA and non-GDM-PLA groups in the third trimester (*p* = 0.0385) (Figure 1B). According to the American College of Obstetricians and Gynecologists, weight gain should be evaluated based on the pre-pregnancy BMI categories (underweight, normal, overweight, and obese) (18). We measured the overall weight gain of each subject by the end of the pregnancy and compared it between the two intervention groups. In the normal-weight category, the mean weight gain in the MI group was 10.2 kg, slightly below the recommended range of 11.5–16 kg. In contrast, the placebo group had a mean weight gain of 11.9 kg, which falls within the recommended range. For the overweight category, both groups exhibited mean weight gains below the lower limit of the recommended range (7–11.5 kg). The MI group had a mean weight gain of 5.5 kg, while the placebo group had a mean of 3.5 kg. In the obese category, the MI group showed a mean weight gain of 2.8 kg, which is notably below the recommended range of 5–9 kg. Conversely, the placebo group had a mean weight gain of 16.7 kg, far exceeding the upper limit of the recommended range. However, the standard deviation shows great interindividual variability, impacting the results’ significance (Table 1).

### 3.2 Lifestyle and physical activity

**Figure 2:**
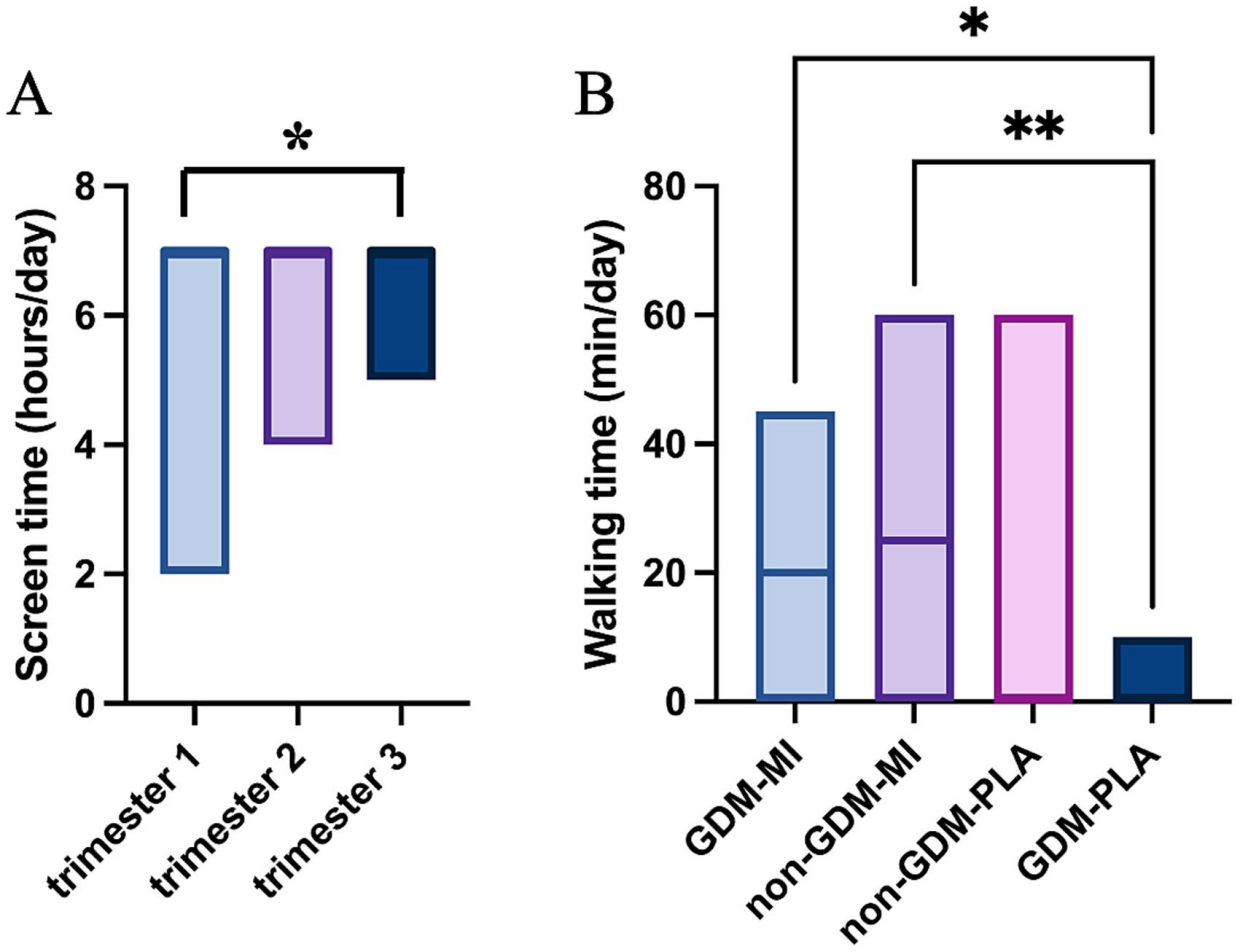
Lifestyle data analysis by using the Kruskal–Wallis test: **(A)** screen time across trimesters; **(B)** walking time across intervention groups; *p < 0.05; **p < 0.01.

Our investigation of the participants’ lifestyle habits showed no differences in physical activity at the baseline, except for a relatively shorter sleeping time (Supplementary Table 1). During the intervention period, we recorded a significant difference in walking time (minutes/day) (*p* = 0.004), with the MI group (both GDM and non-GDM) being more active than PLA. Additionally, screen time (hours/day) showed significant differences between trimesters (*p* = 0.046), reflecting behavioral changes over time and between the two intervention groups (Figure 2 and Supplementary Table 2). It’s worth mentioning that, overall, most of the participants performed insignificant physical activity and did not adhere to the recommended sleeping time across groups (Supplementary Table 2).

### 3.3 Dietary pattern analysis

**Figure 3:**
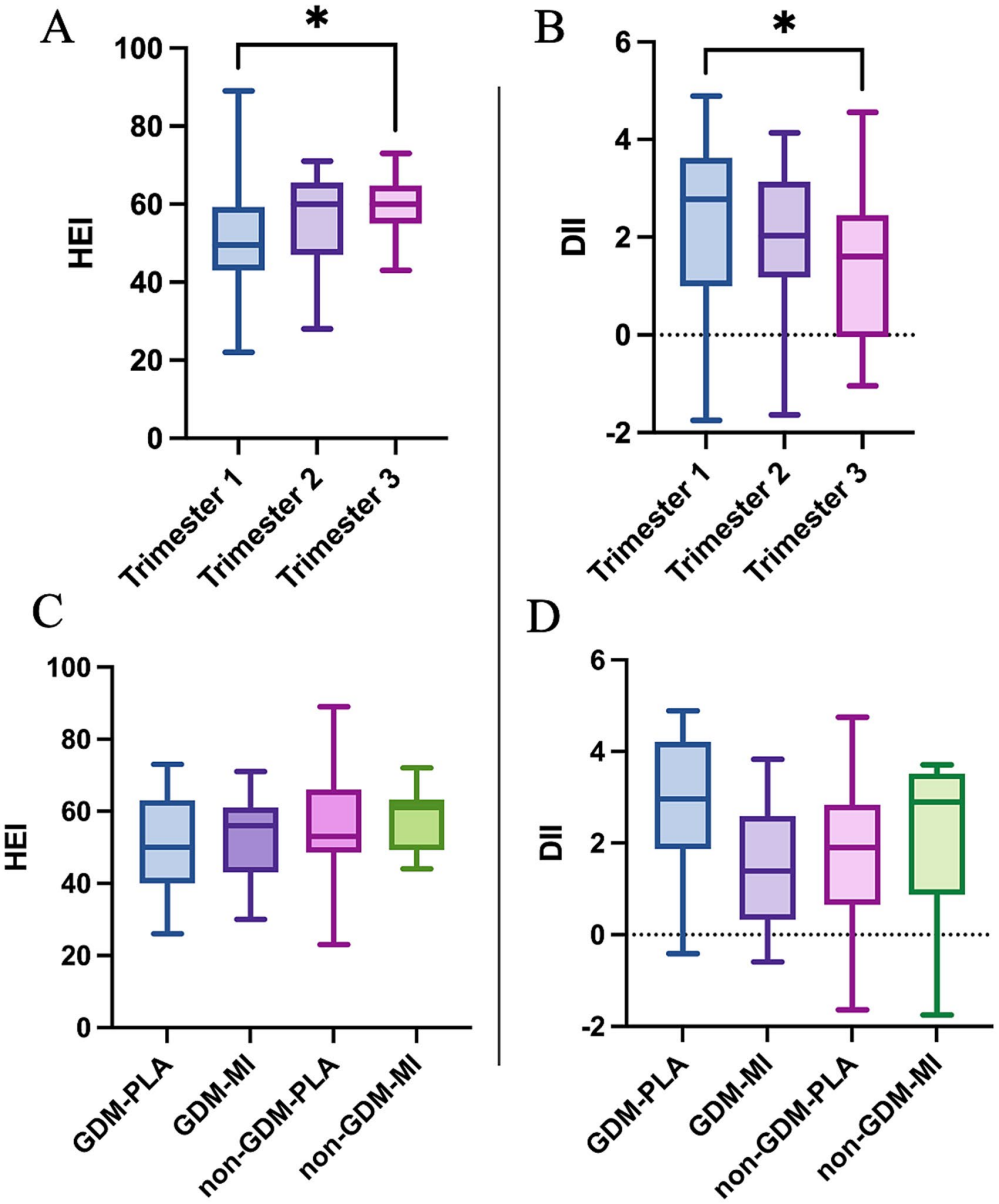
Dietary scores across trimesters: **(A)** HEI; **(B)** DII; and across intervention groups: **(C)** HEI and **(D)** DII compared by using the One-Way ANOVA test; *p < 0.05.

Diet indexes were used to assess the quality of diet at the baseline and across trimesters, with the HEI addressing adherence to the dietary recommendations and the DII measuring the inflammatory effect of certain dietary habits. The HEI score was similar at the baseline to show then a significant improvement in the quality of diet during the three trimesters (*p* = 0.0328), with a major difference between 1st and 3rd trimesters (*p* = 0.0268). The DII follows a similar trend with an high pro-inflammatory diet at the baseline and across pregnancy, which reduced going toward the end of pregnancy (*p* = 0.0462), and in particular when comparing 1st vs. 3rd trimester (*p* = 0.0354) (Figures 3A,B and Supplementary Table 3). We observed that the GDM-MI group adhered more closely to an anti-inflammatory diet compared to the placebo group, but without reaching a statistical difference (Figure 3D), even if no improvement was observed in the HEI (Figure 3C).

### 3.4 Analysis of the food frequency consumption

**Figure 4:**
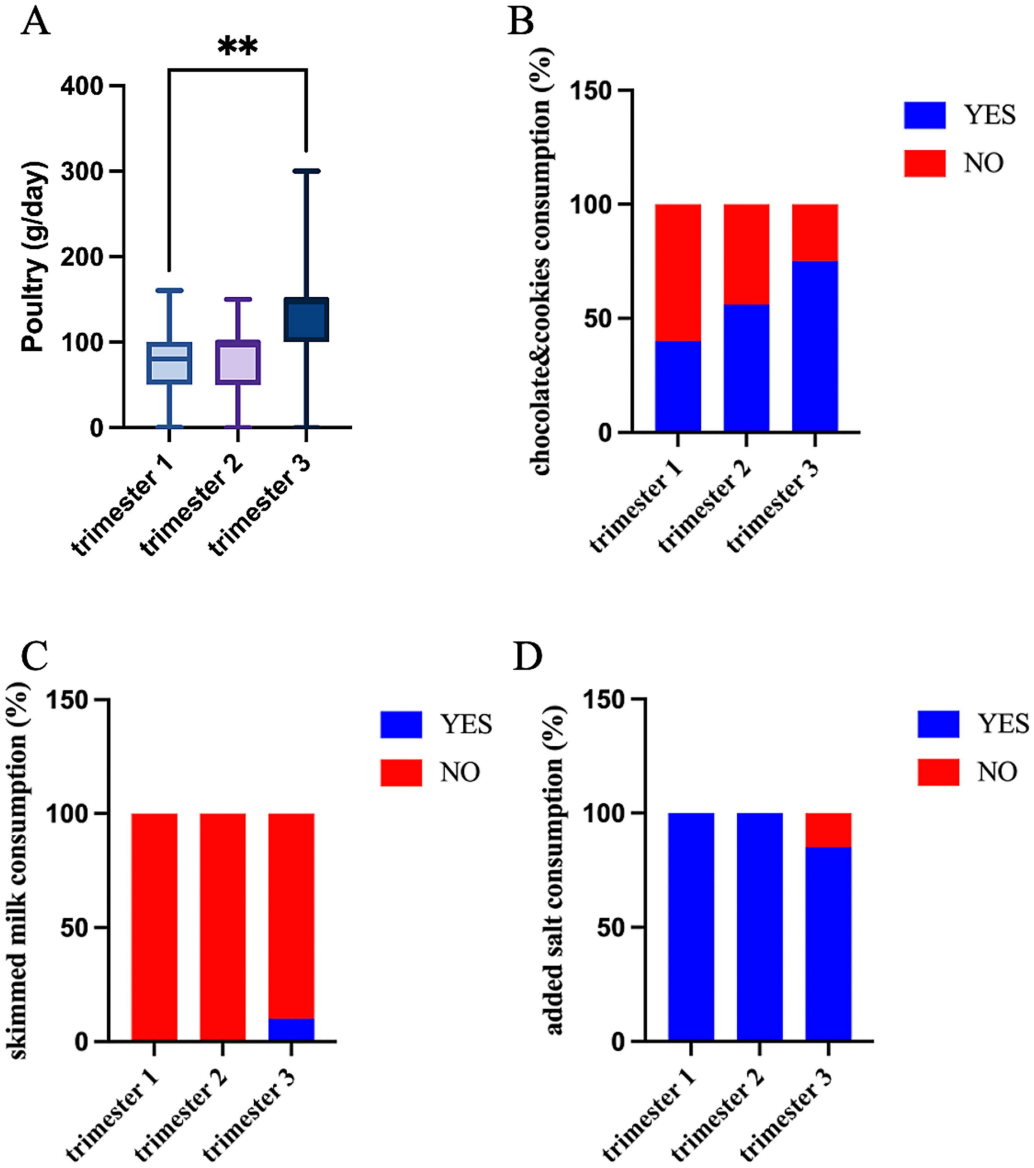
Food intake significantly differs across trimesters: **(A)** poultry quantity (g/day) (Kruskal–Wallis test; *p < 0.05); consumption habits (% of YES/NO) of **(B)** chocolate and cookies; **(C)** added salt; and **(D)** skimmed milk (Fisher’s exact test).

The analysis of the dietary habits did not show any differences in the frequency of food consumption at the baseline (Supplementary Table 3), whether significant differences were identified across pregnancy and between the intervention groups. A larger amount of poultry was consumed daily in trimester three (*p* = 0.013, Figure 4A) along with an increase in the preference for chocolate, cookies, and cakes (*p* = 0.011, Figure 4B). On the contrary, the consumption of added salt decreased in trimester three (*p* = 0.005, Figure 4D) and at the same time, the women started consuming skimmed milk (*p* = 0.032, Figure 4C).

**Figure 5:**
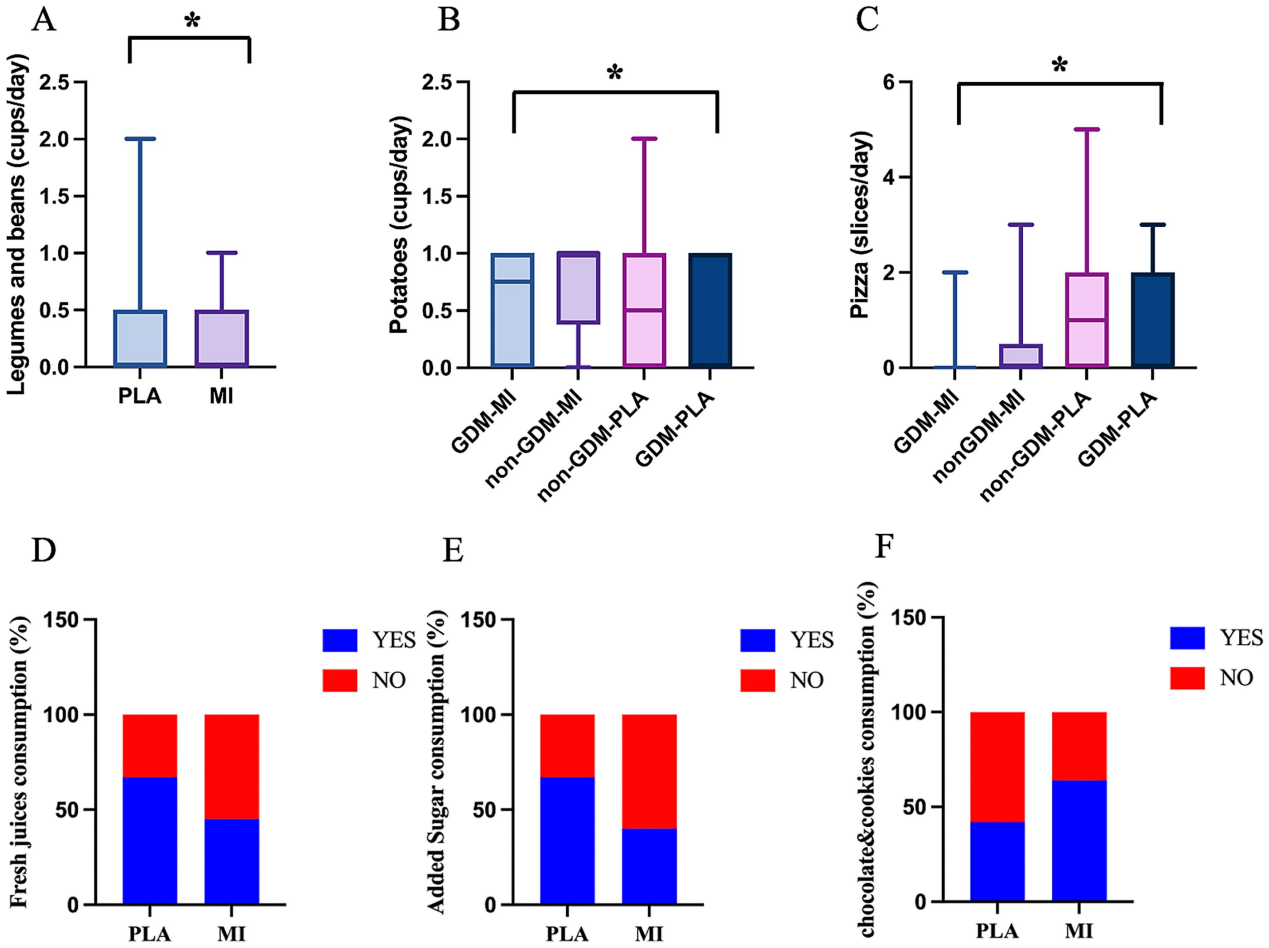
Food intake significantly differs across treatment groups: quantity of **(A)** legumes and beans (cups/week) (Mann–Whitney test); **(B)** potatoes (cups/week) (Kruskal–Wallis test); **(C)** pizza (slices/week) (Kruskal–Wallis test); *p < 0.05; and consumption habits (% of YES/NO) of **(D)** fresh juices; **(E)** added sugar; and **(F)** chocolate and cookies (Fisher’s exact test).

Most importantly, changes in food preferences were also observed between the two treatment arms. In particular, the daily intake of legumes, beans, and tubers was higher in the MI group (*p* = 0.008 and *p* = 0.036, respectively, Figures 5A,B), whereas pizza consumption was higher in the PLA group, both GDM and non-GDM (*p* = 0.019, Figure 5C). Moreover, the chi-square analysis revealed differences in the daily consumption of fresh juices, added sugar, chocolates, and cookies. Participants in the MI group exhibited lower consumption of freshly pressed fruit/vegetable juice (45.2%) compared to the PLA group (66.7%, *p* = 0.044) and sugar (40.5% vs. 66.7%, *p* = 0.014, Figures 5D,E). In contrast, the same group consumed significantly more chocolate, cookies, and cake (64.3%) than those in the placebo group (42.2%, *p* = 0.038, Figure 5F). All these results together suggest that the MI groups follow a healthier diet, with increased consumption of recommended foods (legumes, tubers) and reduced intake of high sugar and fat foods (added sugar, fruit juices, and pizza), except for sweets.

### 3.5 Analysis of the single nutrient intake

**Figure 6:**
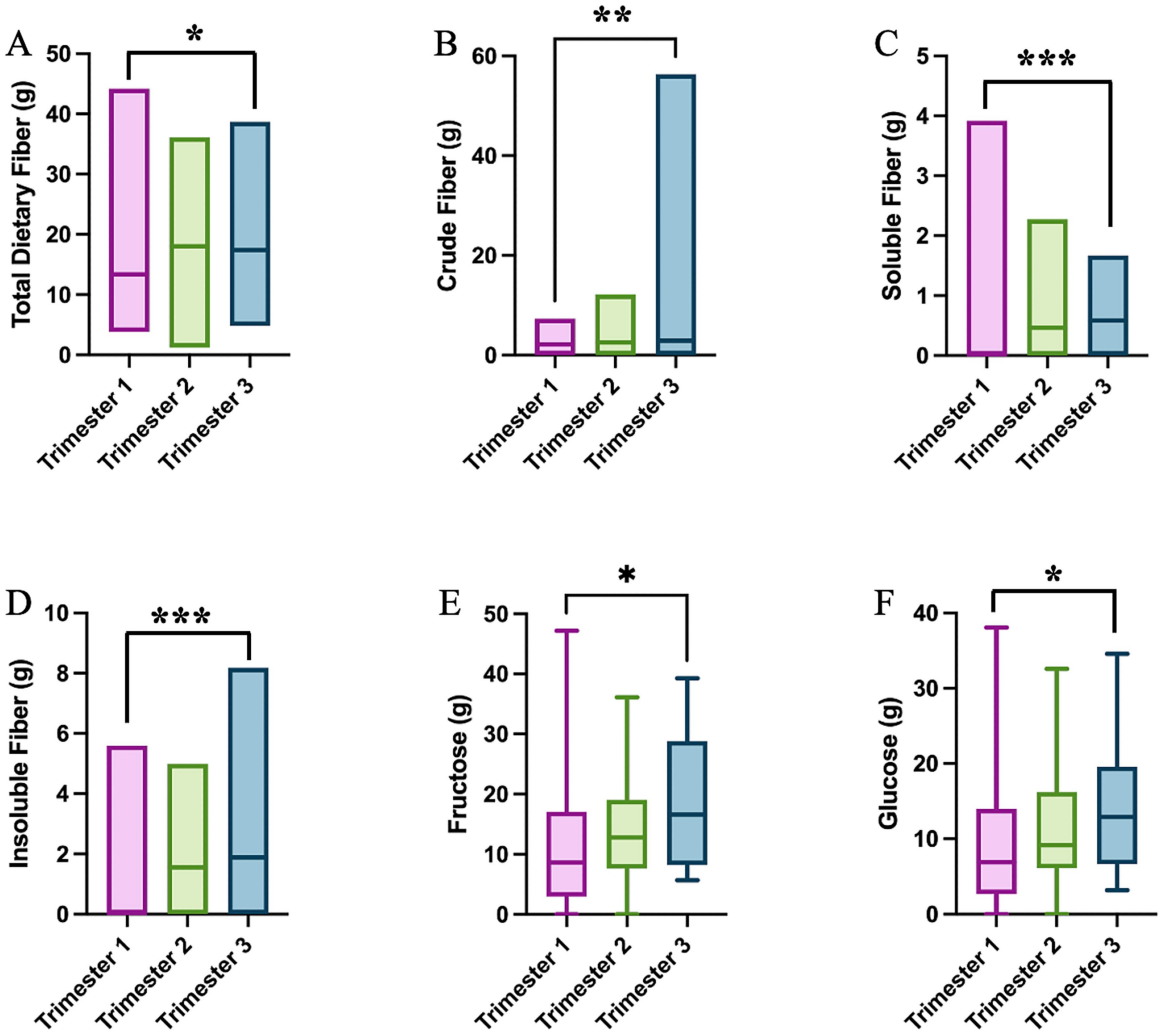
Single nutrient intake significantly differs across trimesters: **(A)** total dietary fiber (One-Way ANOVA test); **(B)** crude fiber (One-Way ANOVA test); **(C)** soluble fiber (One-Way ANOVA test); **(D)** insoluble fiber (One-Way ANOVA test); **(E)** fructose (Kruskal–Wallis test); and **(F)** glucose (Kruskal–Wallis test); *p < 0.05; **p < 0.01; ***p < 0.001.

The daily nutrient intake was computed from the 24HR recalls and compared between MI and PLA groups. We first analyzed the modification in nutrient intake at the baseline and during pregnancy. No differences emerge in nutrient intake between the GDM and non-GDM groups at the baseline (Supplementary Table 4). During pregnancy, instead, the total dietary fiber intake appears to decrease in the second trimester to recover in trimester three (*p* = 0.018, Figure 6A). This is reflected by changes in the subtype of fibers. The intake of crude fiber peaks at trimester three (*p* = 0.005, Figure 6B), whereas the soluble fiber gradually decreases across trimesters (*p* = 0.001, Figure 6C), and insoluble follows shows an opposite behavior, with the highest intake at trimester three (*p* < 0.0001, Figure 6D). A similar trend is shown in the intake of fructose and glucose (*p* = 0.037 and *p* = 0.024, Figures 6E,F). This trend suggests a marked shift in dietary behavior and nutritional requirements as pregnancy progresses, potentially reflecting an increased emphasis on fiber-rich foods during the later stages of pregnancy. The intake of all amino acids, many minerals and vitamins also change during the course of pregnancy (Supplementary Table 5).

**Figure 7:**
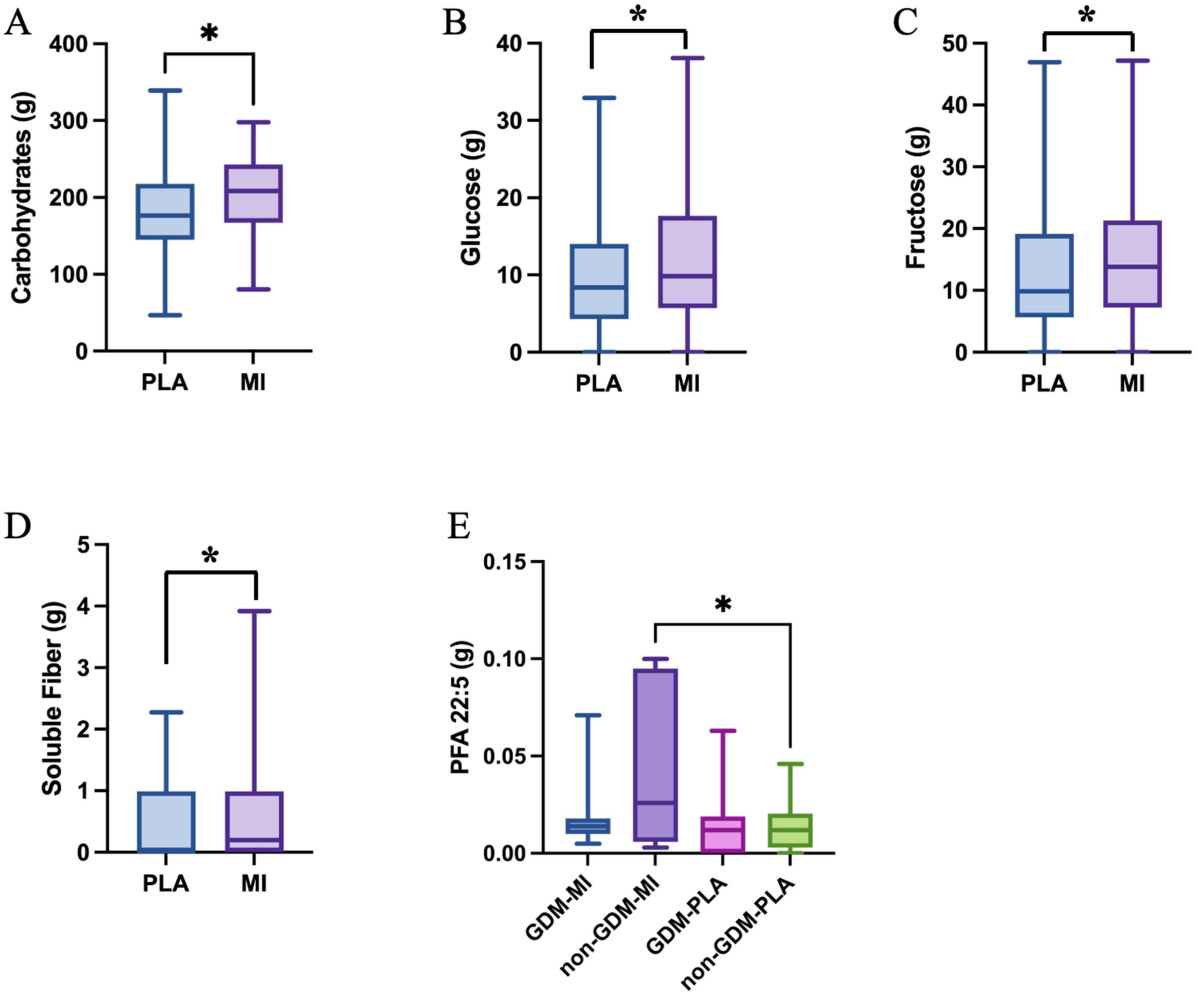
Single nutrient intake significantly differs across treatment groups: **(A)**, Carbohydrate (Mann-Whitney test); **(B)**, Glucose (Mann-Whitney test); **(C)**, Fructose (Mann-Whitney test); **(D)**, Soluble Fiber (One-Way ANOVA test) in women treated with MI vs. PLA; and **(E)**, PFA 22:5 within GDM and non-GDM cases treated with PLA vs MI (One-Way ANOVA test); *p < 0.05.

The nutrient intake also differs between the two intervention groups. Carbohydrate, glucose, fructose and soluble fiber intake are all significantly greater in the MI group compared to the PLA group (*p* = 0.047, *p* = 0.029, *p* = 0.030, and *p* = 0.036, respectively) as shown in Figures 7A–D. The polyunsaturated fatty acid PFA22:5, eicosapentaenoic acid (EPA), showed a significantly higher intake in the non-GDM-MI group compared to both GDM-MI and GDM-PLA and non-GDM groups (*p* = 0.017; Figure 7E).

## 4 Discussion

Myoinositol has been extensively demonstrated to play a role in the prevention of GDM and the treatment of polycystic ovary syndrome and reproductive disorders. It enhances insulin sensitivity and mimics insulin action on metabolic enzymes by affecting the insulin signaling pathway, leading to improved fasting blood glucose levels and reduced glucose fluctuations (10). Multiple studies have explored the effects of MI supplementation in preventing GDM (14, 15, 19). However, the MI effect can be impacted by diet and lifestyle. Our team carried out a randomized double-blinded clinical study to test the effect of MI on pregnant women in Qatar (15), a country with an extremely high incidence of GDM (3). The data showed no differences in the GDM onset between the MI and the placebo arm. As part of this study, we looked for any interference of diet and lifestyle with MI treatment by investigating the dietary habits and lifestyle of pregnant women enrolled in both intervention arms and at each trimester of pregnancy.

With no difference at the baseline, our study confirmed a natural adjustment of the diet during pregnancy and provided detailed information on specific food preferences and nutrient intake changes peculiar to our study population. We observed a trend toward a healthier and less inflammatory diet (increased HEI and decreased DII), mainly due to the increase in high-protein food (poultry), fibers (mainly crude and insoluble), and reduced intake of added salt. An exception is the increased intake of simple sugars (glucose and fructose) and sweets (chocolates, cookies, and cakes), which increased during pregnancy. It’s well recognized that the pregnancy transition across trimesters usually leads to increased appetite and cravings or just increased consumption of certain foods, which can contribute to excessive gestational weight gain and increase the risk of GDM (20). For this reason, diet assessment and caloric restriction are the first line of intervention for GDM (21). Studies have shown promising results of the use of prebiotics and probiotics, plant-based low-protein diets, and the Mediterranean diet as potential therapeutic interventions for the management of GDM (22). In this study, we clearly showed differences in diet preferences, nutrient intake, and physical activity in the MI group compared to the placebo group, which can partially explain the negative results of the myoinositol intervention in reducing the risk of GDM in the population of Qatar. Other factors must be taken into consideration, such as the small study size, the high drop-out rate, and the different ethnicities, and therefore a different genetic background, which are not subjects of this study.

Our findings showed that the PLA group (both GDM and non-GDM) was less active (less walking time/week), gained more weight at the end of the pregnancy, and also consumed more high-fat and high-sugar foods (pizza, added sugar and fresh juices) compared to the MI group, which consume more food high in fibers (beans and tubers). An Iranian study revealed that the intake of fast food, such as pizza, among women of reproductive age was associated with negative impacts on the incidence of gestational diabetes mellitus (GDM) (23). High consumption of simple sugars during gestation may lead to excessive gestational weight gain (GWG) and the development of additional pregnancy problems, including gestational diabetes mellitus (GDM), preeclampsia, and premature delivery (24). A Malaysian study found that consuming fruit juices in early pregnancy—whether homemade or commercial—was moderately correlated with the likelihood of developing GDM. While fruit juices contain beneficial nutrients, the study suggested they do not have a detrimental impact on GDM risk (25), however, the excessive intake of sugar can mask the effect of beneficial nutrients, such as minerals and vitamins (26).

The GDM-PLA groups showed the lowest walking time/week compared to the MI group (both GDM and non-GDM). However, it is noteworthy that the majority of participants engaged in minimal physical activity overall. This low level of physical activity may contribute to the lack of differences observed in other clinical parameters. The analysis of clinical parameters recorded across different trimesters and treatment groups revealed that only fasting glucose levels during the OGTT exhibited statistically significant differences among the treatment groups. Addressing physical activity could be crucial for improving outcomes in future interventions.

Weight gain is a notable outcome in the context of GDM (20). In our study population, a higher weight gain was observed between the first and third trimesters in the PLA group, for both individuals with GDM and those without GDM. These findings confirm the contribution of a healthier diet in managing body weight, which has a tremendous impact on GDM. We can speculate a possible effect of MI as well in controlling the weight gain (with the non-GDM-MI group showing a non-significant trend to a lower weight gain during pregnancy, Figure 1B).

Our study presents several strengths and limitations that inform these findings and their applicability. A key strength lies in its design as a randomized controlled trial (RCT), which minimizes bias and strengthens the validity of the results. Additionally, the longitudinal evaluation of myoinositol’s impact on metabolic parameters across multiple trimesters provides critical insights into the timing and effectiveness of dietary interventions during pregnancy. However, the study’s small sample size limits the generalizability of the findings, as a larger cohort would enhance statistical power and representation. Furthermore, the reliance on self-reported dietary intake introduces potential bias, as inaccuracies in participant recall may affect data reliability.

## 5 Conclusion

Women at risk of GDM taking MI showed a more active lifestyle and a healthy diet in terms of nutrients, food intake, and a trend to a lower inflammatory pattern, with a potential beneficial effect on weight management. The findings support the potential benefits of dietary modifications in combination with myoinositol supplementation as a preventive strategy for GDM. However, further detailed investigations are needed to clarify if there is interference between MI and diet. Future research should also consider larger, more diverse cohorts to validate these findings and explore the long-term effects of dietary and physical activity interventions on pregnant women supplemented with myoinositol. Investigating the integration of structured physical activity programs alongside dietary modifications and supplementation could provide deeper insights into their combined effects.

## Data Availability

The original contributions presented in the study are included in the article/Supplementary material, further inquiries can be directed to the corresponding author.
