# Supplementary material for: Myo-inositol supplementation in gestational diabetes mellitus: is there any interference with diet?
Source: Front Nutr. 2025 Sep 24;12:1623699. doi: 10.3389/fnut.2025.1623699 (PMC12504215; doi:10.3389/fnut.2025.1623699)
Supplement: Supplementary file 1 [file Data_Sheet_1.PDF]

frontiers

Supplementary Material

Supplementary Table 1: Descriptives on lifestyle information at baseline between GDM and non-GDM groups.

Lifestyle habit	*GDM Mean ± SD*	*non-GDM Mean ± SD*	*p-value*
Walking frequency (times/week)	2.67+3.08	1.91+2.21	NS
Walking time (minutes/day)	13.33+18.25	12.73+15.06	NS
Vigorous PA frequency (times/week)	0.00+0.00	0.00+0.00	NS
Vigorous PA time (minutes/day)	0.00+0.00	0.00+0.00	NS
Moderate PA frequency (times/week)	0.00+0.00	0.00+0.00	NS
Moderate PA time (minutes/day)	0.00+0.00	0.00+0.00	NS
Screen time (hours/day)	6.58+0.79	5.91+1.57	NS
Sleeping hours (hours/day)	5.17+1.11	6.00+0.63	**0.041**

Note: The analysis was performed using the One-Way ANOVA test according to data normality.

Supplementary Table ~~1~~2: Descriptives on lifestyle information among the four groups, as per the intervention arm and GDM status.

Lifestyle habit	*GDM-MI Mean ± SD*	*non-GDM-MI Mean ± SD*	*GDM-PLA Mean ± SD*	*non-GDM-PLA Mean ± SD*	*p-value*
Walking frequency (times/week)	1.9 ± 2.1	3.1 ± 2.7	1 ± 1.8	1.9 ± 2.4	0.928
Walking time (minutes/day)	20 ± 16.15	21.67 ± 18.7	2.73 ± 4.7	10.7 ± 11.7	**0.004**

Vigorous PA frequency (times/week)	0 ± 0	0 ± 0	0.09 ± 0.3	0.33 ± 1.5	0.364
Vigorous PA time (minutes/day)	0 ± 0	0 ± 0	1.82 ± 6.03	1.43 ± 6.5	0.690
Moderate PA frequency (times/week)	0.07 ± 0.3	0 ± 0	0 ± 0	0.1 ± 0.4	0.364
Moderate PA time (minutes/day)	3 ± 11.6	0 ± 0	0 ± 0	2.9 ± 13.1	0.784
Screen time (hours/day)	6.4 ± 1.3	6.7 ± 0.7	7 ± 0	6.43 ± 0.9	0.317
Sleeping hours (hours/day)	6 ± 0.8	5.7 ± 1.2	6.2 ± 0.8	5.8 ± 0.9	0.539

Note: The analysis was performed using the One-Way ANOVA test according to data normality.

Supplementary Table 3: Descriptives on food frequency and dietary score information at baseline between GDM and non-GDM groups.

Food frequency and dietary scores	*GDM Mean ± SD*	*non-GDM Mean ± SD*	*p-value*
DII $^{\$}$	2.31+1.78	2.43+1.82	NS
HEI $^{\$}$	52.4+12.3	47.9+15.4	NS
Full fat milk (cup)	0.47+0.77	0.59+0.73	NS
Low fat milk (cup)	0.25+0.45	0.22+0.41	NS
Skimmed milk (cup)	0.00+0.00	0.00+0.00	NS
Soya milk (cup)	0.00+0.00	0.00+0.00	NS
Sweetened milk (cup)	0.00+0.00	0.00+0.00	NS

Almond milk (cup)	0.00+0.00	0.045+0.150	NS
Water (litr)	1.35+0.58	1.022+0.284	NS
Tea/coffee (cup)	0.33+0.49	0.73+0.647	NS
Soft drinks(cup)	0.08+0.28	0.18+0.405	NS
Freshly pressed fruit/vegetable juice (cup)	0.500+0.522	0.682+0.462	NS
Alcohol (cup)	0.00+0.00	0.00+0.00	NS
Bread (slice)	1.66+0.98	1.864+0.323	NS
Cereals (cup)	0.385+0.475	0.295+0.458	NS
Rice (cup)	0.79+0.582	0.76+0.560	NS
Biscuit, pancake (slice)	3.66+4.63	1.45+1.63	NS
Legumes, beans (cup)	0.15+0.29	0.13+0.32	NS
Fresh vegetables (cup)	0.83+0.71	0.81+0.60	NS
Cooked vegetables (cup)	0.62+0.48	0.71+0.39	NS
Fruits (cup)	1.83+1.52	1.45+0.82	NS
Nuts (g)	21.72+33.53	20.31+30.55	NS
Tubers, potatoes, sweet potatoes (cup)	0.75+0.45	0.63+0.45	NS
Noodles, pasta (cup)	0.45+0.49	0.59+0.49	NS
Red meat (g)	50.83+58.06	35.45+37.77	NS
Poultry (g)	96.67+62.86	80.00+39.74	NS
Fish, seafood (g)	51.67+68.99	42.73+49.21	NS

Processed food (slice)	0.00+0.00	1.00+2.40	NS
Cheese (tbs)	0.00+0.00	0.22+0.60	NS
Yogurt (cup)	0.70+0.45	0.81+0.40	NS
Eggs (n)	1.25+0.8	1.27+0.78	NS
Pizza (slice)	0.92+1.6	0.73+0.78	NS
Olive oil (TBS)	0.83+0.71	0.90+0.53	NS
Corn oil (TBS)	0.41+0.66	0.36+0.67	NS
Sunflower oil (TBS)	0.42+0.79	0.00+0.00	NS
Canola oil (TBS)	0.00+0.00	0.00+0.00	NS
Sesame oil (TBS)	0.00+0.00	0.00+0.00	NS
Other oils	0.08+0.28	0.03+0.09	NS
Butter	0.27+0.44	0.30+0.45	NS
Ghee	0.16+0.57	0.09+0.30	NS
Lard	0.00+0.00	0.09+0.30	NS
Salt (TSP)	1.16+0.38	1.18+0.60	NS
Sugar (TSP)	0.70+0.810	0.59+0.49	NS
Fast food (time/week)	0.75+0.86	0.64+0.67	NS
Fried potatoes (time/week)	1.17+1.99	0.91+0.83	NS
Chocolate, cookies, cake, donuts (time/week)	1.58+2.61	3.00+3.09	NS

Note: The analysis was performed using the One-Way ANOVA test according to data normality.

Supplementary Table 4: Descriptives on nutrient intake at the=baseline between GDM and non-GDM groups.

Nutrient	*GDM Mean ± SD / Median (IQR)*	*non-GDM Mean ± SD / Median (IQR)*	*p-value*
Kilocalories (kcal) [#]	1485.51(2005.01)	1412.14(1746.64)	NS
Protein (g) [#]	55.53(138.74)	64.30(56.94)	NS
Carbohydrate (g) [#]	170.47(229.94)	210.80(217.21)	NS
Fat, Total (g) [#]	46.52(75.57)	55.03(97.00)	NS
Alcohol (g) [#]	0.0(0.10)	0.0(17.01)	NS
Cholesterol (mg) [#]	214.43(651.39)	290.83(513.17)	NS
Saturated Fat (g) [#]	18.64(38.24)	18.56(30.16)	NS
Monounsaturated Fat (g) [$]	12.61+7.124	18.33+12.776	NS
Polyunsaturated Fat (g) [#]	7.11(27.43)	8.21(15.48)	NS
SFA 4:0 (g) [#]	0.35(0.61)	0.23(0.82)	NS
SFA 6:0 (g) [#]	0.21(0.37)	0.13(0.60)	NS
SFA 8:0 (g) [#]	0.16(0.42)	0.09(0.41)	NS
SFA 10:0 (g) [#]	0.38(0.82)	0.22(0.83)	NS
SFA 12:0, Lauric (g) [#]	0.39(1.80)	0.21(0.95)	NS
SFA 14:0 (g) [#]	1.37(2.32)	0.75(3.01)	NS
SFA 16:0, Palmitic (g) [#]	6.75(10.88)	6.86(8.92)	NS
SFA 17:0 (g) [$]	0.04+0.041	0.04+0.023	NS

SFA 18:0, Stearic (g) $	2.65+1.664	2.94+0.948	NS
SFA 20:0 (g) #	0.01(0.13)	0.04(0.25)	NS
SFA 22:0 (g) $	0.03+0.064	0.05+0.078	NS
MFA 14:1 (g) $	0.02+0.033	0.02+0.032	NS
MFA 16:1 (g) $	0.47+0.276	0.49+0.213	NS
MFA 18:1, Oleic (g) $	11.17+6.748	13.72+6.302	NS
MFA 20:1 (g) $	0.09+0.051	0.12+0.099	NS
MFA 22:1 (g) $	0.00+0.004	0.01+0.005	NS
PFA 18:2, Linoleic (g) $	7.52+6.625	8.61+4.510	NS
PFA 18:3, Linolenic (g) $	0.925+0.983	0.82+0.339	NS
PFA 18:4 (g) $	0.00+0.001	0.00+0.008	NS
PFA 20:4 (g) $	0.11+0.091	0.15+0.097	NS
PFA 20:5, EPA (g) $	0.01+0.039	0.01+0.042	NS
PFA 22:5 (g) $	0.01+0.010	0.01+0.019	NS
PFA 22:6, DHA (g) $	0.04+0.066	0.05+0.074	NS
Trans Fatty Acid (g) $	0.22+0.319	0.38+0.417	NS
Sodium (mg) $	2032.23+1064.383	2200.51+1195.15	NS
Potassium (mg) #	2070.35(4815.96)	1966.52(3492.55)	NS
Chloride (mg) $	39.08+135.388	330.42+992.109	NS
Vitamin A (RE) $	555.80+692.983	566.66+441.362	NS

Vitamin A (IU) $	4440.47+6986.508	4131.93+4906.56	NS
Total Carotenoid (RE) $	0.00+0.00	0.00+0.00	NS
Beta-Carotene (mcg) $	4566.36+5630.994	2643.60+2858.83	NS
Alpha-Carotene (mcg) $	591.06+897.708	691.28+838.672	NS
Lutein (+ Zeaxanthin) (mcg) $	4827.61+8639.793	1093.23+1001.34	NS
Beta-Cryptoxanthin (mcg) $	103.01+147.627	153.33+205.880	NS
Lycopene (mcg) $	1209.21+3104.434	1043.12+2140.54	NS
Vitamin C (mg) $	88.62+99.745	74.50+73.027	NS
Calcium (mg) #	645.77(1151.93)	778.33(1046.64)	NS
Iron (mg) #	10.30(16.55)	8.26(9.92)	NS
Vitamin D (ug) (mcg) $	1.97+1.840	2.80+1.565	NS
Vitamin D (IU) $	100.99+69.698	130.52+62.609	NS
Vitamin E (mg) $	0.32+0.518	0.68+1.153	NS
Vitamin E (IU) $	0.42+0.626	1.00+1.721	NS
Vitamin E (Alpha-Tocopherol) (mg) #	4.82(8.19)	4.91(8.84)	NS
Thiamin (mg) #	0.91(2.28)	1.13(1.44)	NS
Riboflavin (mg) $	1.20+0.517	1.43+0.58	NS
Niacin (mg) #	12.70(61.71)	15.57(14.16)	NS
Niacin Equivalent (mg) $	15.82+12.032	14.56+3.54	NS
Pyridoxine (Vitamin B6) (mg) #	1.40(5.18)	1.68(2.77)	NS
Folate (Total) (mcg) #	282.39(1240.99)	292.10(338.83)	NS

Cobalamin (Vitamin B12) (mcg) #	1.73(5.10)	2.04(3.22)	NS
Biotin (mcg) #	9.21(29.44)	14.84(39.840)	NS
Pantothenic Acid (mg) #	2.29(6.35)	3.61(4.31)	NS
Vitamin K (mcg) $	168.28+300.95	54.21+36.39	NS
Phosphorus (mg) #	739.00(1835.86)	792.26(1260.41)	NS
Iodine (mcg) $	0.41+1.44	10.93+25.66	NS
Magnesium (mg) #	235.66(506.55)	213.28(284.76)	NS
Zinc (mg) $	5.66+3.261	6.33+3.326	NS
Copper (mg) #	0.89(2.46)	0.88(1.50)	NS
Manganese (mg) #	1.76(4.12)	1.68(3.50)	NS
Selenium (mcg) #	60.01(272.72)	83.42(111.34)	NS
Fluoride (mcg) $	2354.54+4687.29	2365.26+3732.36	NS
Chromium (mg) #	0.01(0.07)	0.00(0.04)	NS
Molybdenum (mcg) $	7.15+11.162	9.99+11.47	NS
Choline (mg) $	261.08+224.131	289.90+150.16	NS
Dietary Fiber, Total (g) $	17.14+11.311	14.97+8.62	NS
Soluble Fiber (g) $	0.40+0.714	0.31+0.57	NS
Insoluble Fiber (g) $	0.82+1.154	0.78+1.25	NS
Crude Fiber (g) $	2.18+2.160	1.82+1.46	NS
Sugar, Total (g) #	53.77(84.32)	88.14(110.41)	NS

Glucose (g) #	7.23(25.68)	10.19(31.93)	NS
Galactose (g) $	0.59+1.331	1.31+2.489	NS
Fructose (g) #	9.17(33.67)	16.72(46.32)	NS
Sucrose (g) #	11.64(22.45)	12.85(56.23)	NS
Lactose (g) $	3.47+4.755	10.51+12.031	NS
Maltose (g) $	0.50+0.625	1.11+0.86	NS
Tryptophan (mg) #	280.80(978.20)	338.12(357.15)	NS
Threonine (mg) #	950.73(3382.82)	1180.57(1256.84)	NS
Isoleucine (mg) #	1167.33(3797.91)	1382.91(1518.21)	NS
Leucine (mg) #	2047.50(6368.72)	2382.21(2928.51)	NS
Lysine (mg) #	1446.32(6549.29)	1920.53(2291.84)	NS
Methionine (mg) #	577.63(1970.16)	720.62(873.44)	NS
Cystine (mg) #	418.91(999.16)	477.79(490.88)	NS
Phenylalanine (mg) #	1249.43(3515.17)	1531.16(1510.62)	NS
Tyrosine (mg) #	946.94(2768.01)	1046.68(1399.96)	NS
Valine (mg) #	1473.91(4335.44)	1743.72(2156.32)	NS
Arginine (mg) #	1267.49(5290.49)	1689.08(2032.66)	NS
Histidine (mg) #	660.16(2597.24)	777.44(901.96)	NS
Alanine (mg) #	1124.29(4276.45)	1431.23(1711.36)	NS
Aspartic Acid (mg) #	2425.50(7797.33)	2780.88(2818.14)	NS
Glutamic Acid (mg) #	5547.33(12486.78)	6240.99(6153.88)	NS

Glycine (mg) #	810.36(3353.02)	1093.65(1160.89)	NS
Proline (mg) #	1719.59(3237.37)	1952.37(3178.84)	NS
Serine (mg) #	1377.81(3672.04)	1721.68(1974.83)	NS
Caffeine (mg) $	40.85+48.903	32.81+39.441	NS
Folate (DFE) (mcg) #	366.09(1323.09)	413.85(406.60)	NS
Vitamin A (RAE) (mcg) $	657.43+01.543	492.97+285.397	NS
Kilojoules (kj) #	5905.12(7310.67)	6077.85(8391.84)	NS
Sum of Trans Fat and Sat Fat (g) #	5.86(26.88)	6.32(27.41)	NS
Salt (g) $	5.07+2.661	5.50+2.986	NS
Added Sugars (g) $	4.41+11.748	13.20+21.983	NS
Folic Acid (mcg) $	44.76+29.16973	93.58+74.77421	0.048
Beta Tocopherol (mg) $	0.018+0.02425	0.09+0.08742	0.009
Gamma Tocopherol (mg) $	0.58+0.83137	2.25+2.18567	0.022
Delta Tocopherol (mg) $	0.06+0.08967	0.50+0.60412	0.020
Alpha Tocotrienol (mg) $	0.04+0.038	0.13+0.159	NS
Food Folate (mcg) $	288.51+348.865	154.20+101.522	NS
Betaine (mg) $	11.02+15.948	34.50+36.512	NS
Phytosterols (mg) $	15.00+23.199	18.14+17.436	NS
SFA 15:0 Pentadecanoic acid (g) $	0.03+0.043	0.03+0.034	NS
SFA 24:0 Lignoceric Acid (g) $	0.01+0.038	0.02+0.044	NS

Total isoflavones (mg) $	0.01+0.028	0.06+0.072	0.047

Note: The analysis was performed using the One-Way ANOVA test ($) or Kruskal-Wallis' test (#), according to data normality.

Supplementary Table 25: Descriptives on Significant nutrient intakes by trimesters (only the significant nutrients are shown) by using the One-Way ANOVA test ($) or Kruskal-Wallis' test.

Nutrient	*Trimester 1 Mean ± SD / Median (IQR)*	*Trimester 2 Mean ± SD / Median (IQR)*	*Trimester 3 Mean ± SD / Median (IQR)*	*p-value*
MFA 18:1, Oleic (g) $	14.8 ± 8.7	17.1 ± 9.3	21.6 ± 12.4	0.043
PFA 22:5 (g) $	0.0103 ± 0.01	0.02 ± 0.02	0.02 ± 0.02	0.037
Lycopene (mcg) $	901.4 ± 1962.8	3846.04± 6115.2	4862.6 ± 5233.9	0.002
Niacin Equivalent (mg)$	16.2 ± 10.4	18.9 ± 10.5	25.8 ± 18.1	0.023
Dietary Fiber, Total (g) $	15.4 ± 8.4	16.6 ± 8.7	22.1 ± 8.9	0.018
Soluble Fiber (g) $	0.4 ± 0.8	0.3 ± 0.5	1.01 ± 0.6	0.001
Insoluble Fiber (g) $	0.8 ± 1.3	0.8 ± 1.3	2.7 ± 1.7	0.000
Crude Fiber (g) $	2.3 ± 1.7	2.8 ± 2.3	7.9 ± 12.8	0.005
Folic Acid (mcg) $	92.2 ± 73.9	123.2 ± 72.3	60 ± 46.2	0.011
Beta Tocopherol (mg) $	0.07 ± 0.09	0.07 ± 0.08	0.16 ± 0.16	0.008
Gamma Tocopherol (mg) $	1.3 ± 1.6	2.2 ± 2.9	3.13 ± 2.6	0.016
Phytosterols (mg) $	19.0 ± 20.2	26.9 ± 25.9	52.8 ± 29.9	0.000
SFA 15:0 (g) $	0.03 ± 0.03	0.07 ± 0.09	0.04 ± 0.05	0.033

Chromium (mg) #	0.012(0.04)	0.04(0.04)	0.05(0.04)	0.002
Biotin (mcg) #	10.7(16.6)	13.5(23.6)	24.7(19.7)	0.002
Potassium (mg) #	1966.7(1117.3)	2074.8(607.7)	2746.2(1366.6)	0.008
Aspartic Acid (mg) #	2475.4(1717.2)	2789.5(2242.9)	4359.9(5455.1)	0.017
Arginine (mg) #	1470.4(1338.8)	1908.7(1706.5)	2759.9(3241.7)	0.019
Glycine (mg) #	935.4(902.8)	1387.1(1053)	1794.9(2638.3)	0.023
Lysine (mg) #	1581.1(1617.5)	2270.6(2236.7)	3281.3(4591.6)	0.023
Glucose (g) #	6.9(10.4)	8.4(8.3)	15.1(12)	0.024
Tyrosine (mg) #	954.9(824.2)	1342.4(799.2)	1604.9(1885.3)	0.025
Alanine (mg) #	1280.2(1166.2)	1675.9(1273.7)	2307.1(3028.2)	0.026
Histidine (mg) #	674.9(557.3)	922.8(901.3)	1169.1(1615.02)	0.027
Threonine (mg) #	964.01(933.6)	1363.4(1017.6)	1833.02(2377.8)	0.027
Magnesium (mg) #	223.8(123.3)	213.3(119.9)	272.9(88.3)	0.029
Isoleucine (mg) #	1247.7(1052.2)	1594.2(1220.6)	2140.01(2697.3)	0.031
Phenylalanine (mg) #	1346.5(971.5)	1632.1(1011.4)	2075.2(2059.6)	0.033
Leucine (mg) #	2134.8(1795.2)	2770.5(2071.9)	3564.6(4278.5)	0.033
Cystine (mg) #	434.4(332.6)	514.9(348.4)	611.4(627.2)	0.034
Serine (mg) #	1365.04(1109.8)	1805.8(1510.6)	2336.1(2100.2)	0.035
Valine (mg) #	1521.9(1346.6)	1910.8(1430.02)	2569.3(2687.3)	0.036
Methionine (mg) #	588.1(559.2)	805.3(629.8)	1068.1(1497.5)	0.036

Fructose (g) #	8.2(15.2)	12.3(9.4)	17.6(17.7)	0.037
Selenium (mcg) #	77.9(57.4)	92.8(67.2)	96.9(37.4)	0.043
Pantothenic Acid (mg)	3.3(1.8)	3.6(2)	4.3(1.9)	0.045
Tryptophan (mg) #	308.3(256.7)	414.1(290.2)	482.9(652.9)	0.046
Glutamic Acid (mg) #	5925.5(4231.6)	6914.3(5399.8)	8175.1(8613.2)	0.047

Note: The analysis was performed using the One-Way ANOVA test ($) or Kruskal-Wallis' test (#), according to data normality.
